# A prospective observational study of lifestyle behaviors and biomarkers to promote cardiometabolic health in healthcare workers during the COVID-19 pandemic

**DOI:** 10.3389/fmolb.2026.1714752

**Published:** 2026-04-01

**Authors:** Mary S. McCarthy, Zachary T. Colburn, Laurel H. Gillette, Nancy S. Hodge, Ling-Hong Hung, Bryce Fukuda, Daniel Rhee, Joshua Sakai, Ka Yee Yeung

**Affiliations:** 1 Madigan Army Medical Center, Tacoma, WA, United States; 2 The Geneva Foundation, Tacoma, WA, United States; 3 School of Engineering and Technology, University of Washington Tacoma, Tacoma, WA, United States; 4 Department of Computer Science and Engineering, University of California Santa Cruz, Santa Cruz, CA, United States

**Keywords:** cardiometabolic health, digital biomarkers, healthcare workforce, lifestyle behaviors, precision health

## Abstract

**Introduction:**

Nutrition, physical activity, and sleep deficits contribute to diminished health status in healthcare workers (HCWs). The purpose of this study was to focus attention on HCWs by incorporating precision health approaches to address lifestyle behaviors believed to influence immune health, cardiometabolic status, and wellbeing.

**Methods:**

In this 1-year prospective observational study, a digital dashboard was created to post a health promotion video, targeted health messages, and personal health data accessible to each participant. Activity-sleep trackers were provided to capture actual step and sleep activity. Lifestyle questionnaires, serum biomarkers, body composition by bioelectrical impedance analysis, and blood pressure were obtained at four timepoints; gene expression was measured at three timepoints. Genes linking host immune response to key nutrients were isolated for correlational analyses with lifestyle behaviors. Participants received a comprehensive summary of their personal results at 12 months. Primary outcome was participant engagement.

**Results:**

104 HCWs enrolled between July and October 2021. At 12 months, 60% of participants were high adopters for device-wear time and daily step threshold. Daily steps declined by 831 (±3,441) steps per day, t statistic −1.742, *p* = 0.09 after 1 year. Insomnia Severity Index results suggested persistent subclinical insomnia with a mean score of 8.1 (±6.1) at 12 months. Thirteen genes had a moderate correlation with steps, *r* (49) = 0.29–37, sleep, *r* (56) = 0.27–0.37, distance, *r* (61) = 0.28, and Insomnia Severity Index, *r* (56) = 0.30–0.38, *p* = NS, after Benjamini–Hochberg adjustment.

**Conclusion:**

Participant engagement may have contributed to favorable behavior change in the first 6 months, but this was not sustained at 12 months. Novel strategies are needed to boost engagement for longer studies, promote HCW wellness to optimize immune and cardiometabolic health, and abate chronic disease progression.

## Introduction

“To continue to provide uninterrupted, quality care, the healthcare workforce - human beings - must be empowered and encouraged to take care of themselves” (Nanda et al., 2017).

On 11 March 2020, the World Health Organization (WHO) declared the novel coronavirus outbreak of SARS-CoV-2, or COVID-19, a global pandemic (World Health Organization WHO, 2020). Healthcare workers (HCWs) then, and now, face a dynamic and unpredictable threat while caring for patients with the highly transmissible virus or related conditions in emergency rooms, intensive care units, and general medical floors in every U.S. hospital. The coronavirus pandemic exposed debilitating inadequacies in our healthcare systems while also opening the eyes of Americans to the impressive competence, dedication, compassion, and resilience of HCWs who remained steadfast in their oath to care for the sick (American Nurses Association ANA, 2015). Four years post-pandemic, the continued emergence of variants that evade the immune system creates uncertainty and anxiety despite high levels of fully vaccinated communities and an adequate supply of personal protective equipment. Healthcare professionals, both military and civilian, are physically, psychologically, and morally exhausted. While generally viewed as a high-risk occupation for post-traumatic stress disorder, fatigue, burnout, anxiety, and depression (Nanda et al., 2017; Melnyk et al., 2021; Hassamal et al., 2021), the unprecedented duration of the pandemic and its sequelae that includes the dwindling resources (e.g., manpower, emergency room beds) to support care delivery demands, has contributed to a further decline in the health and resilience of the medical workforce (Melnyk et al., 2022; Shreffler et al., 2020).

Among 6,271,313 laboratory-confirmed COVID-19 cases reported during 1 January 2020–12 October 2021, by CDC, 7.02% (440,044) were in HCWs and ultimately resulted in a total of 1,469 HCW deaths (Centers for Disease Control and Prevention CDC, 2023; Lin et al., 2022). Other causes of death, such as suicide, are not included in these numbers. Given the significant role played by frontline HCWs in caring for patients with both acute and long-term COVID-19, as well as interacting with family members, it is not surprising that reports of emotional exhaustion and mental health symptoms have exceeded pre-pandemic levels (Melnyk et al., 2022; Shreffler et al., 2020). In August 2020, the National Academy of Medicine’s Action Collaborative on Clinician Wellbeing and Resilience began to raise public awareness of the vulnerabilities borne by the healthcare workforce (National Academy of Medicine NAM, 2020). The report called for urgent action at the organizational level to safeguard the health and wellbeing of clinicians risking their lives every day to treat and heal patients. While the psychological toll was more acute, the underlying medical conditions of HCWs of all ages became a lower priority yet continued to place the HCW at risk for chronic non-communicable disease. Reports during the COVID-19 pandemic stated that over 89% of the HCWs had at least one underlying condition with the most common being obesity (72%), hypertension (41%), and diabetes (31%); all conditions that require ongoing treatment and monitoring (Centers for Disease Control and Prevention CDC, 2020). Research conducted post-pandemic continues to report low levels of physical activity and disrupted sleep as some of the suboptimal health behaviors expressed by healthcare professionals (Holtzclaw et al., 2020; Ramot et al., 2024). Melnyk et al. (2022) reported that physicians and nurses do a poor job of self-care, and as a result, dismiss the potential benefits of healthy lifestyle behaviors. One outcome of the poor mental and physical health is the higher likelihood of making medical errors compared to nurses in better health (Melnyk et al., 2021). There is empirical evidence for other signs of impaired functioning associated with extended and variable shifts as well as the related insomnia, to include needle stick and musculoskeletal injuries, patient safety incidents, poor teamwork, low productivity and job performance, and absenteeism (Stimpfel, 2020). In addition, the rotating shift work, and lengthy periods of night shift in particular, lead to circadian misalignment resulting in disruptions in neuroendocrine and immune system health along with links to cancer, mood disorders, cardiovascular disease, and metabolic dysfunction (Stimpfel, 2020; Morris et al., 2016). While research connects sleep disturbances and increased levels of pro-inflammatory biomarkers Interleukin-1, Interleukin -6, and tumor necrosis factor, results have been inconsistent and often conflicting (Irwin et al., 2016; Besedovsky et al., 2019; Bjorvatn et al., 2020). The Centers for Disease Control and Prevention (CDC) support the recommendation of the Joint Consensus Statement of the American Academy of Sleep Medicine and Sleep Research Society for 7 or more hours of sleep per night for adults, but no more than 9 h, for optimal health (Watson et al., 2015; Centers for Disease Control and Prevention CDC, 2022a). Finally, one might expect that HCW would see themselves as role models and educators for patients struggling with health-promoting behaviors but evidence suggests otherwise with HCW having increased body mass index and greater risk of developing obesity than the general public (Roskoden et al., 2017). In addition to the known favorable effects on cardiometabolic health, physical activity may induce short-term immune adaptations through anti-inflammatory effects on visceral fat mass reduction (Belanger et al., 2022). A wearable electronic activity tracker can be an effective tool for self-surveillance and self-regulation thus promoting behavior change for improved physical activity and wellbeing (Liau et al., 2018; Hebden et al., 2014; Kirk et al., 2019; Gavin et al., 2018). In general, experts believe that “HCW wellbeing supports improved patient-clinician relationships, a high-functioning care team, and an engaged and effective workforce” (National Academy of Medicine NAM, 2020). The purpose of this study was to focus attention on HCWs by incorporating precision health approaches to address lifestyle behaviors believed to influence immune health, cardiometabolic status, and wellbeing.

## Materials and methods

### Setting and participants

In August 2021, we initiated a 6-month prospective, longitudinal, observational study in a single level II military medical center in the Pacific Northwest with a professional workforce of about 2,500 men and women. Mid-study, we received additional funding and an extension allowing for a full year of observation through September 2022. A staff weekly bulletin announcing the research study was the source for 80% of the inquiries and subsequent enrollments. Poster boards and flyers in high visibility areas and word-of-mouth led to enrollment of the remaining 20% of the sample.

Any interested HCW (registered nurse, licensed practical nurse, physician, dentist, physician assistant, pharmacist, dietitian, psychologist, social worker, chiropractor, or technician) who was eligible to receive care at the military medical center and would remain employed by the organization for the next year, was invited to participate. We excluded those planning to leave their position (resign, relocate, or terminate military service), and those unable or unwilling to consent to the study protocol such as wearing an activity tracker, submitting peripheral blood samples on three occasions, completing online questionnaires, and attending three in-person appointments. Additionally, volunteers currently using a tanning bed or taking doses of vitamin D supplements over 600 IU/day had to agree to discontinue tanning and limit vitamin D to 600 IU/day for 2 months prior to using phototherapy. For anyone planning to use phototherapy, he/she must not have a current diagnosis of skin cancer or sarcoidosis, or light allergies or sensitivities including actinic prurigo, polymorphous light eruption, solar urticaria, protoporphyria, photodermatitis, xeroderma pigmentosum, lupus erythematosus, actinic dermatitis, or UV-sensitive syndrome. The Institutional Review Board (IRB) of the military medical center approved the study (IRB # 221049). Each participant signed and dated a consent document with embedded details of the Health Insurance Portability and Accountability Act of 1996 (HIPAA) prior to any data collection and before continuing for the additional 6 months. This document also contained the signature and date of the team member conducting the informed consent process.

#### Data collection

An in-person appointment was scheduled with the Principal Investigator (PI) and Project Director (PD) for baseline, 6 months, and 12 months. Each appointment lasted about 60 min. An email check-in occurred at 3 months and participants received links to electronic surveys. At in-person appointments, the participant completed questionnaires, body composition and blood pressure measurements, online account setup with instructions for the wrist-worn activity and sleep tracker and study dashboard access, and a viewing of the health promotion video.

##### Demography

The demographic questionnaire was investigator-developed for participants to self-report 1) date of birth, 2) sex assigned at birth, 3) race, 4) ethnicity, 5) marital status, 6) education level attained, 7) employment status (active duty military or government civilian), 8) military rank or grade, 9) occupation, 10) relevant personal and family history, 11) underlying medical conditions, 12) medications, 13) moderate and vigorous physical activity levels, 14) activity restrictions, 15) current tobacco use, 16) current alcohol consumption, 17) typical hours of sleep on weekdays and weekends, 18) frequency of regular sun exposure, 19) sunscreen use, 20) skin tone classification, 21) number of days of missed work due to illness in previous year, and 22) vaccination status.

##### Participant engagement

The study team created a toolkit of evidence-based resources to support HCWs in their efforts to modify unhealthy behaviors contributing to a decline in physical and immune health. The toolkit resources intended to raise awareness and provide feedback included: 1) biomarker and gene expression analysis, 2) body composition via bioelectrical impedance analysis (BIA), and blood pressure, 3) an activity and sleep tracker, 4) personalized digital dashboard, 5) targeted health messaging, 6) Ultraviolet (UV) B phototherapy, and 7) a health promotion video. Levels of engagement (also called adoption) with the various toolkit components were assessed at 2 timepoints using a Visual Analog Scale, 6 months and 12 months. Participants placed a mark on a horizontal line marked for 0–4 inches; score was recorded as 0 to 4. Based on the published literature, we designated high engagement as 70% wear-time for the activity/sleep tracker based on data extraction from the Google Application Programming Interface (API) for each participant. Additional thresholds were set for steps, sleep, dietary intake according to published guidelines; details for each variable follow.

##### Cardiometabolic assessment

Circulating plasma biomarkers selected for the metabolic assessment included fasting blood glucose, total cholesterol, high and low-density lipoprotein cholesterol, triglycerides, vitamin C, vitamin D, and zinc. Physical measurements included height in inches rounded to the nearest 0.5 inch using a portable stadiometer (Seca 213, Chico, CA), weight in pounds, lean body mass, fat mass, and percent body fat, with calculated body mass index and basal metabolic rate via InBody 270 BIA (InBody USA, Cerritos, CA). We measured blood pressure (BP) using a digital monitor and universal adult cuff (Omron® Healthcare, Inc., Lake Forest, IL.). We adhered to the American Heart Association (AHA) guidelines (Flack and Adekola, 2020) for obtaining BP by performing two readings on the same arm at least 1 min apart which were then averaged and for establishing normal ranges for both systolic and diastolic readings. For atypical, elevated readings, BP was taken a third time prior to the end of the appointment. Heart rate was annotated as well.

##### Gene expression

At the same time as the venipuncture for the metabolic assessment, blood was collected for targeted genes which included 773 on the Host Immune Response panel (nCounter®), 12 internal reference genes for data normalization, and 30 genes custom-ordered and meticulously selected from published Genome-Wide Association Studies (GWAS) or high quality experimental trials for their role in nutrient metabolism. The whole blood sample was drawn into the PreAnalytixX PAXgene® (Qiagen, Ann Arbor, MI, United States) Blood RNA tube.

Samples were processed manually in batches for RNA extraction. RNA concentration and quality was quantified by Qubit (ThermoFisher, Waltham, MA, United States) and normalized to 10 ng/μL. The hybridization reaction to attach CodeSet and Reporter Plus oligos was performed at 65 °C for 24 h on a QIAamplifier 96 (Qiagen) thermocycler. Gene expression detection was performed on the NanoSprint Profiler nCounter instrument.

##### Physical activity

Each participant received a digital activity tracker (Fitbit Flex™) with two wristbands and a charging cable upon enrollment. Trackers were distributed to promote more walking at work, before and/or after shifts, on breaks, during lunch, or when using the staff walking trail maps available onsite. The device tracked and displayed daily steps, distance walked, calories burned, and minutes of sleep. We requested that participants wear the tracker continuously except during water activities. The PI or PD emphasized the Center for Disease Control and Prevention (CDC) recommendations of 150–300 min of moderate to vigorous or 75 min of vigorous activity each week (Centers for Disease Control and Prevention CDC, 2022b; 2018 Physical Activity Guidelines Adviso ry Committee, 2018) with 2 days of resistance training for maximal cardiometabolic and immune health (Centers for Disease Control and Prevention CDC, 2022b; Shao et al., 2021). A threshold of 7,000 steps or more per day was established to designate a high adopter or high level of engagement for participants (Palu et al., 2021).

##### Dietary intake

We used the 2014 Block Food Frequency Questionnaire (FFQ; Nutrition Quest, Berkeley, CA, United States) to assess dietary intake (Block et al., 1990). The food list for the FFQ was developed from analysis of two waves of National Health and Nutrition Examination Survey (NHANES) dietary recall data, 2007–2008 and 2009–2010. The nutrient and food group analysis database was developed from the United States Department of Agriculture’s Food and Nutrient Database for Dietary Studies (Bowman et al., 2008), the Food Pyramid Equivalents Database (Bowman et al., 2008), and the Nutrient Database for Standard Reference (Haytowitz et al., 2019). There is no validation publication for the 2014 Block FFQ. The food and beverage list includes 127 items with customization to assess vitamins C and D, zinc, and n-3 fatty acids. Participants completed an online survey on 4 occasions with a reference period of the previous 30 days; survey data were compiled by Nutrition Quest and sent to the study PI. A brief summary report was printed for participants at the time of each administration; the study PI, PD, or Registered Dietitian (RD) provided feedback and evidence-based recommendations regarding energy intake and macro- and micronutrient consumption based on age, body size, physical activity levels, and current evidence for boosting immune health (Linus Pauling Institute, 2020; Lichtenstein et al., 2021).

The Nutrition Quest team calculated the Dietary Inflammatory Index (DII) score (Shivappa et al., 2014) and the Healthy Eating Index (HEI)-2015 score (Krebs-Smith et al., 2018) from each FFQ administration. The DII is based on forty-five pro- and anti-inflammatory food parameters identified in the literature. When fit to this composite global database, the DII score of the maximally pro-inflammatory diet was +7.98, the maximally anti-inflammatory DII score was – 8.87 and the median was +0.23.

The HEI-2015 has two component categories, adequacy (nine components) and moderation (four components) with scores calculated based on established minimum and maximum standards (Krebs-Smith et al., 2018). Food intake meeting or exceeding the standard receives the highest score for the adequacy component, and food intake meeting the standard or lower receives the maximum number of points for the moderation component. Each component is weighted equally at 10 points with total score calculated from the sum of individual component scores with a maximum potential score of 100. The HEI-2015 was developed so that a standardized approach could be used to compare dietary quality across the general population. For this reason, the HEI-2015 uses a density approach with each component scored per 1,000 kilocalorie consumed (Hiza et al., 2018).

##### Sleep

Sleep data in minutes were extracted from the tracker data repository created using Google (Fitbit) API. Participants also completed an online Insomnia Severity Index (ISI) (Bastien et al., 2001), a 7-item self-report questionnaire assessing the nature, severity, and impact of insomnia. The recall period was the previous 2 weeks, and the dimensions evaluated were: difficulty falling asleep, difficulty staying asleep, early morning awakenings, sleep pattern dissatisfaction, interference of sleep difficulties with daytime functioning, noticeability of sleep problems by others, and distress caused by the sleep difficulties. A 5-point Likert scale is used to rate each item (e.g., 0 = no problem; 4 = very severe problem), yielding a total score ranging from 0 to 28 with 0–7 meaning no clinically significant insomnia, 8–14 meaning subthreshold insomnia, 15–21 meaning moderate to severe clinical insomnia, and 22–28 indicating severe clinical insomnia (Bastien et al., 2001). Sleep messaging, developed from the American Academy of Sleep Medicine, Sleep Research Society, and the CDC (Besedovsky et al., 2019; Watson et al., 2015; Centers for Disease Control and Prevention CDC, 2022a), was provided to participants to assist with achieving ISI score <8 (no clinically significant insomnia) at 6 and 12 months. The daily goal for sleep was 7 or more hours (Watson et al., 2015; Centers for Disease Control and Prevention CDC, 2022a); the threshold was set at 7 h (420 min) to distinguish high and low engagement/adoption.

##### Personalized digital dashboard

The bioinformatics team (KYY, LHH, DR, JS) designed and implemented an interactive dashboard that automated data download from the activity tracking device and FFQ. Specifically, the interactive dashboard used Javascript and the Google (Fitbit) API to systematically retrieve sleep and activity data from all participants around the clock and select dietary intake data from the FFQ on each administration. In addition, the PI and PD provided diet and sleep questionnaire scores, body composition and BP data, and accessible laboratory testing results for cardiometabolic health for each individual participant to update personal dashboard displays. Results were uploaded to the interactive dashboard, deployed on Microsoft Azure, so that participants had access to their personal health data anywhere, anytime, during the study. The dashboard was organized into four tabs: general information, sleep, activity, and nutrition.

##### Targeted health messaging

Participants had access to data-driven health messaging as a form of personalized feedback. Similar to the organization of the dashboard, the health messages were divided into the following categories: general, activity, sleep, and nutrition. The general messages were uniform across all participants, while messages in the other categories were triggered by individual health behaviors or activities which did not meet pre-specified thresholds. For example, baseline data for specific nutrients queried in the FFQ including vitamin C, D, zinc, cholesterol, sweets, calories divided into protein, carbohydrate and fat, and fat categorized as total, monounsaturated, and polyunsaturated were posted. If intake of any nutrient was outside the normal range per the Dietary Guidelines for Americans, a message with strategies to optimize consumption of the nutrient in the diet was displayed on the participant’s dashboard.

Educational outreach using the dashboard was continually updated as data became available.

##### Ultraviolet B (UVB) phototherapy

Wall-mounted phototherapy devices were available as an optional resource for low 25(OH)D status. Interested participants were educating on using the device no more than twice a week, once on the torso front side and once on the back. Participants established a secure, password-protected account with the manufacturer’s platform (Solius, Inc. Bainbridge Island, WA); visits were tracked for safety purposes, frequency of use, and dose delivered. The manufacturer provided technical oversight for safety, troubleshooting, and report preparation. The device and detailed instructions for use were accessible to participants 7 days a week, 24 h a day in a private research office onsite.

##### Health promotion video

The study team created a 7-min health promotion video on location that would maximize the viewer’s experience when learning about targeted, yet simple, lifestyle modifications to reduce risk for cardiometabolic disease. A script of evidence-based recommendations focused on dietary intake, physical activity, sleep, and general wellbeing.

Participants viewed the YouTube video during the enrollment visit and were encouraged to access it using the dashboard portal for the duration of the study. Number of video viewings was self-reported at 6 and 12 months.

### Outcomes

Primary outcome was participant engagement as measured by the activity tracker wear-time at 6 and 12 months.

Secondary outcomes included daily steps and minutes of sleep from the activity-sleep tracker, adoption of toolkit resources measured by VAS score at 6 and 12 months, and HEI-2015 score assessed using the FFQ at Baseline and 12 months.

### Analysis

Descriptive statistics were used to summarize sample characteristics, health behaviors, and body composition; all analyses were performed, and figures were created using the R version 4.1.2 (R Core Team, 2021). Data from the activity and sleep tracker were examined for extreme outliers and excluded if determined to be from activity device error. Categorical variables were compared using Fisher’s exact test or Chi-square test (depending on sample sizes). Pearson’s correlation was used for pairs of continuous variables and the ANOVA or t-test was used when integrating categorical and continuous variables.

Gene expression analyses were performed using the nCounter Analysis System (NanoString Technologies, Inc. Seattle, WA, United States). Gene expression data were analyzed via multiple linear regression using NanoString’s nSolver 4.0 software. Statistical significance was established using alpha of 0.05; significance was adjusted for multiple testing using the Benjamini–Hochberg false discovery rate (FDR) technique at the time of the final analysis (Yoav and Hochberg, 1995).

## Results

### Sample demographics

Between July–October 2021,104 HCWs from 12 disciplines were enrolled. At 6 months, attrition was 14%, and at 12 months 20% of the extended cohort was lost to relocation and dropouts. This population of HCWs in one military medical center in the Pacific Northwest is representative of the diverse workforce of active-duty military and government civilians in most medical centers throughout the Military Health System. This sample was highly educated with 50% reporting post-graduate education, mostly physician specialties, and they were generally healthy with 20% reporting hypertension on enrollment, and only half of the participants reported missing more than 1 day of work each year (see Table 1). We analyzed the demographics of the remaining cohort at Time 4 to identify potential differences between study completers and non-completers; the only demographic with significance (*p* < 0.05) was marital status with more married participants completing the study; 44 versus 33, Fisher’s exact test *p* = 0.024 (see Table 2).

**TABLE 1:** Demographics at baseline (N = 104).

Variable	n	%^a^
Male	30	29
Female	74	71
Caucasian, non-Hispanic	85	82
Status – Active military	45	43
Married	77	74
Post-graduate education	52	50
Occupation		
RN	56	54
MD	16	15
Underlying medical conditions		
Overweight (BMI >24.9)	73	70
Hypertension	21	20
Vitamin D insufficiency	19	18
Tobacco use		
Never	83	80
Alcohol use		
2–4 times/week	39	37
2–4 times/month	25	24
Never	16	15
Days of missed Work/year >1	50	48

^a^ Not all percents add up to 100 due to multiple-response option.

RN, registered nurse; MD, medical doctor.

**TABLE 2:** Demographics at time 4^a^.

Variable	Completers n = 52	Non-completers n = 52
Male	13	17
Female	39	35
Caucasian, non-Hispanic	36	33
Status – Active military	18	27
Married^b^	44	33
Post-graduate education	30	22
Occupation		
RN	28	28
MD	8	6
Underlying medical conditions		
Overweight (BMI >24.9)	37	40
Hypertension	9	10
Vitamin D insufficiency	10	7
Tobacco use		
Never	42	40
Alcohol use		
2–4 times/week	12	11
2–4 times/month	12	13
Never	10	6
Days of missed work/Year - 1 or more	28	24

^a^ Time 4 = 12 months; RN: registered nurse; MD: medical doctor.

^b^ Only significant variable, Fisher’s exact test *p* = 0.024.

### Participant engagement

On average 59% of participants were highly engaged wearing the device 70% of the time at 3 months; 44% met this threshold at 6 months, and 59% met this threshold at 12 months.

Participants were queried regarding adoption of various components of the toolkit; BIA body composition and biomarker analysis earned the highest scores at 3.46 and 3.44 out of 4.0, respectively, 12 months after enrollment (timepoint 4) (see Table 3). The personalized dashboard and health messaging finished 7th and 8th place, respectively. The feedback from participants upon completion of the study was that sending out email messages with the educational content would have been better received and more likely seen and utilized. Attrition of 50% occurred by 12 months leaving 52 participants completing the study. While it is not uncommon to see high attrition in studies involving active-duty military participants due to the highly mobile and transient nature of the workforce, this high attrition may bias engagement and outcome estimates.

**TABLE 3:** Evaluation of participant engagement with 9 toolkit resources at 12 months (n = 52).

Toolkit resource	Visual analog scale score^a^
(2) BIA body composition	3.46
(1) Biomarker and gene expression analysis	3.44
Overall toolkit satisfaction	3.34
(3) Activity tracker	3.20
(6) UVB phototherapy	2.90
(3) Sleep tracker	2.82
(7) Educational video	2.40
(4) Personalized dashboard	1.80
(5) Health messaging	1.70

^a^ Visual Analog Scale 0–4 inches with score 0–4; BIA, bioelectrical impedance analysis; UVB, Ultraviolet B.

**FIGURE 1:**
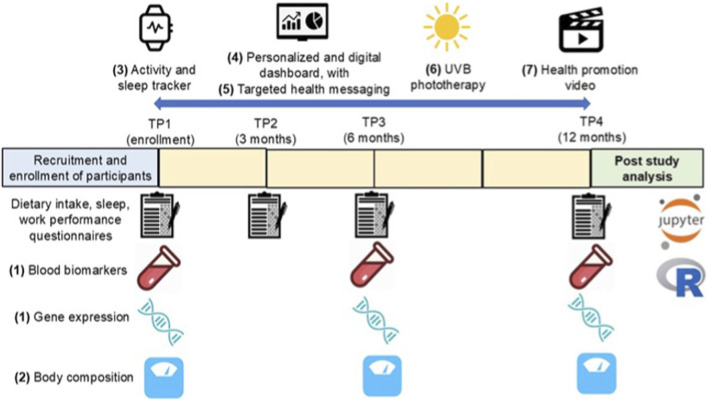
Study measurement timeline. Legend: Toolkit components 1-7 identified in parentheses.

The numbers in parentheses correspond to Figure 1 toolkit components.

Engagement by participants was also examined as a factor in whether their health status improved over time. Specifically, a participant was considered as “stayed healthy” if they met the engagement threshold of at least 70% wear-time for the activity-sleep tracker, and as “stayed unhealthy” otherwise. For each of 12 selected measurements shown in Table 4, we calculated the number of participants with “improved health” (switching from “unhealthy” at baseline to “healthy” at timepoint 4), and the number of participants with “worsened health” (switching from “healthy” at baseline to “unhealthy” at timepoint 4). After calculating the number of participants in each category, we computed the odds ratio and p-values using Fisher’s Exact Test. Table 4 shows that 5 such measurements (steps, ISI, Omega-3, BMI, and percent body fat) achieved significant *p*-values (*p* < 0.001) meaning statistically significant associations between participant engagement, the activity-sleep tracker, and these 5 measurements were observed. The observed changes may reflect temporal, contextual, or pandemic-related factors rather than toolkit exposure alone.

**TABLE 4:** Health status based on high and low participant engagement for 12 selected measurements at timepoint 4 versus Baseline (n = 52)^a^.

Measurement	Stayed healthy^b^	Improved health	Worsened health	Stayed unhealthy^c^	*p*-value#	Odds ratio
Steps	30	5	6	11	<0.001	10.34
Sleep	23	7	14	8	0.36	1.85
ISI	20	9	4	19	<0.001	10.00
Physical activity	18	10	11	13	0.26	2.09
Vitamin C	11	12	7	22	0.09	2.82
Vitamin D	17	11	7	17	0.03	3.65
Zinc	37	6	6	3	0.18	3.00
Omega-3 FA	11	2	6	33	<0.001	27.33
Fiber	22	4	11	15	0.003	7.18
Blood pressure	17	7	7	21	0.001	6.96
BMI	26	3	1	22	<0.001	152.12
% body fat	23	1	5	23	<0.001	91.37

^a^ 12 months; Steps and sleep data from activity tracker; Insomnia Severity Index (ISI) and Physical activity data from questionnaires.

^b^ Stayed healthy: met threshold/high engagement (70% wear time).

^c^ Stayed unhealthy: did not meet threshold/low engagement #Significance *p* < 0.05.

### Cardiometabolic health

**FIGURE 2:**
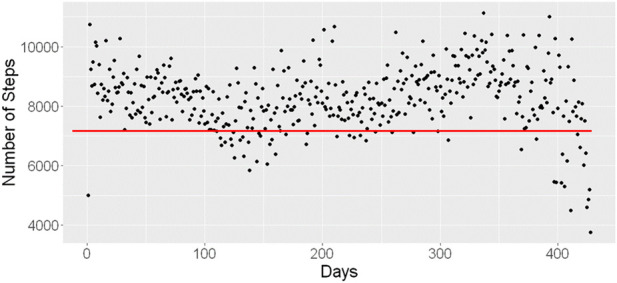
Median steps per day per participant remaining in the study at 12 months (n = 52). Legend: Red line set at threshold of 7,000 steps per day.

There was no significant change in systolic BP for males from Baseline to 12 months or in diastolic BP for males from Baseline to 12 months. Female participants, as a group, met the American Heart Association guidelines for both systolic and diastolic BP throughout the study. Steps initially increased and then began to steadily decline at 6 months with this trend continuing until the study was completed. Participant (n = 52) device data at 12 months revealed a mean decline of 831 (±3,441) steps per day, t statistic −1.742, *p* = 0.09 (see Figure 2); overall physical activity in minutes/week experienced a decline which was significant at 6 months but not significant at 12 months.

Standard cardiometabolic parameters were included in this study (see Tables 5, 6). At 12 months, serum vitamin D increased significantly by 3.68 (11.64) ng/dL, *p* = 0.03 and FBG decreased significantly by 3.33 (7.56) mg/dL, *p* < 0.001.

**TABLE 5:** Baseline biomarkers by gender. All participants enrolled in the study are included.

Variable	Male mean (SD) n = 30	Female mean (SD) n = 74	Reference value/Range
Height (inches)	69.1 (2.7)	64.2 (3.2)	—
Weight (pounds)	193.8 (30.8)	156.4 (28.6)	—
Body mass index	28.6 (4.4)	26.5 (4.7)	≤24.9 kg/m^2^
Body fat (%)	25 (7.7)	32.9 (9.3)	Males <20%
			Females <30%
Lean mass	143.0 (18.7)	103.1 (14.9)	—
Systolic BP	128.6 (8.6)	116.1 (14.2)	120 mmHg
Diastolic BP	81.7 (8.6)	77.0 (9.4)	80 mmHg
Blood glucose	98.2 (8.6)	97.8 (9.0)	<109 mg/dL
Total cholesterol	184.7 (25.8)	184.7 (39.1)	<200 mg/dL
HDL-C	64.1 (18.7)	62.9 (16.4)	Males >40 mg/dL
			Females >50 mg/dL
LDL-C	120.3 (32.0)	110.5 (32.8)	<99 mg/dL
Triglycerides	104.9 (25.9)	85.4 (50.6)	<150 mg/dL
Vitamin D	36.1 (13.9)	37.0 (14.1)	≥30 ng/dL
Vitamin C	1.0 (0.47)	1.1 (0.55)	0.4–2.0 mmol/L
Zinc	85.7 (7.7)	86.1 (15.0)	44–115 mcg/dL

BP, blood pressure; HDL-C, high density lipoprotein cholesterol; LDL-C, low density lipoprotein cholesterol; mg, milligrams; mcg, micrograms; dL, deciliter; ng, nanograms; mmol, millimole; mm, millimeter; Hg, mercury.

**TABLE 6:** Dietary intake and biomarker change from Baseline to Time 4^a^.

Measurement	n	Mean change	sd	t statistic	*p*-value**
Vitamin C (FFQ)	52	59.94	339.20	1.274	0.21
Vitamin D (FFQ)	52	8.99	31.78	2.040	0.046
Zinc (FFQ)	52	−1.21	18.86	−0.463	0.65
Fiber (FFQ)	52	−2.17	10.71	−1.459	0.15
DII (FFQ)	52	−0.702	3.02	−1.286	0.20
HEI-2015 (FFQ)	52	−1.11	10.1	−0.644	0.52
Blood glucose	52	−3.33	7.56	−3.240	<0.001
Total cholesterol	52	2.09	27.15	0.566	0.57
Triglycerides	52	6.98	41.20	1.245	0.22
LDL cholesterol	52	−0.69	24.59	−0.205	0.84
HDL cholesterol	52	0.00	7.97	0.000	1.00
Cholesterol/HDL ratio	52	0.43	2.65	1.180	0.24
Vitamin C (blood)	35	−0.12	0.39	−1.676	0.10
Vitamin D (blood)	50	3.68	11.64	2.236	0.03
Zinc (blood)	44	−6.82	16.54	−2.735	0.009

^a^ 12 months; FFQ: food frequency questionnaire; LDL: low density lipoprotein; HDL: high density lipoprotein.

**Significance = *p* < 0.05.

### Gene expression analysis

Gene expression data were collected at Baseline, 6 months, and 12 months. Through an exploratory gene expression analyses of over 800 genes, we identified 13 genes with moderate and significant correlations with lifestyle behaviors however, after the Benjamini–Hochberg (BH) procedure for False Discovery Rate, correlations were no longer significant. Five genes were categorized as nutrient-responsive, meaning related to dietary intake, and 8 genes were categorized as immune-responsive meaning related to immune health. Table 7 lists the correlations of the 13 genes that showed significant correlations initially (*p*-value <0.05) with the corresponding study variable (sleep, ISI, steps or distance) but failed to show significance after BH adjustment. Participants were presented with a graphical report of gene expression changes at timepoint 3 and timepoint 4 with an emphasis on the exploratory and hypothesis-generating approach to the analysis.

**TABLE 7:** Selected gene, diet, lifestyle, and biomarker relationships from Baseline to Time 4^a^

Gene	Gene category	Study variable	n	Correlation	95% CI lower bound	95% CI upper bound	*Adj p* value
*IL20RB*	NIR	Sleep	56	−0.372	−0.579	−0.121	0.334
*GC*	Nutrient metabolism	Sleep	56	−0.366	−0.574	−0.115	0.334
*CYP27B1*	Nutrient metabolism	Sleep	56	−0.354	−0.564	−0.100	0.334
*FGR*	NIR	ISI	49	−0.375	−0.594	−0.105	0.334
*FYN*	PIR; NIR; T cell signaling	Steps	49	−0.373	−0.592	−0.103	0.334
*HCK*	NIR	ISI	49	−0.347	−0.572	−0.073	0.472
*SYK*	NIR	Steps	49	0.330	0.054	0.560	0.559
*FURIN*	NIR	Steps	49	0.320	0.043	0.552	0.559
*TNF*	PIR; NIR; T cell signaling	Distance	61	−0.281	−0.498	−0.032	0.559
*AMY2A*	Nutrient metabolism	Steps	49	−0.313	−0.546	−0.035	0.559
*FTO*	Nutrient metabolism	ISI	49	0.306	0.027	0.541	0.575
*IL12B*	PIR; NIR	Steps	49	0.297	0.017	0.533	0.607
*VTN*	Nutrient metabolism	Sleep	56	−0.272	−0.499	−0.010	0.607

CI: confidence interval; ISI: insomnia severity index; NIR: negative inflammatory response; PIR: positive inflammatory response.

a Baseline to 12 months.

### Sleep

Sleep data revealed an average of 460 (±58) minutes or 7.7 h per night (see Figure 2), over the 12-month study, although anecdotally, participants perceived a decrease in sleep time, more frequent awakenings, and poor sleep quality. Devices captured a mean decrease of 10 (±201) minutes, t statistic −0.368, *p* = 0.71 between Baseline and 12 months (data not shown). Weekday hours of sleep reported were evenly divided between 4–6 (48.5%) and 7–8 (47.5%) hours, yet weekend hours of sleep reported were 7 or more hours for 80% of participants. The mean (SD) of the Insomnia Severity Index (ISI) suggested persistent subclinical insomnia with a Baseline score of 9.12 (5.9) decreasing to 8.72 (5.4) at 6 months, and to 8.1 (6.1) at 12 months, *p* = NS across all timepoints.

### Dietary intake

Results from the FFQ revealed no increased dietary intake of vitamin D or any other targeted nutrient (vitamin C, n-3 FA, fiber, and zinc) at 12 months. Three related dietary components, total calories, grams of carbohydrate, and grams of saturated fat were significantly lower from baseline to 12 months as reported on the FFQ (see Table 8). The DII and the HEI-2015 were calculated from the 2014 Block FFQ results on 4 occasions. Results show no significant difference with DII mean (SD) at Baseline of −0.603 (3.02) and at 12 months of 0.098 (3.42), t statistic −1.288, *p* = 0.20. Similarly, the HEI-2015, a secondary outcome, did not show a significant difference over time remaining at a median score of 65.2 (10.1). It is important to mention that there is currently no contemporary validation reference for the 2014 Block FFQ leaving its use in this population and setting without established reliability and validity.

**TABLE 8:** Dietary intake of nutrients from FFQ Baseline to 12 months (n = 52).

Dietary component	Timepoint	Mean	SD	Target	*p* value
Calories	1	1898.97	703.16	Men: ∼2,500 kcals	0.025
	4	1,608.53	598.59	Women: ∼2000 kcals	
Protein (gms)	1	76.70	28.68	10%–20% of total calories	0.074
	4	66.88	26.81		
Carbohydrate (gms)	1	205.00	80.43	50%–60% of total calories	0.039
	4	173.69	71.93		
Saturated fat (gms)	1	26.50	12.02	<7% of calories	0.044
	4	22.15	9.62		
Vitamin C (mg)	1	97.74	63.19	200–400 mg	0.609
	4	91.10	68.93		
Vitamin D (mcg)	1	5.03	3.27	15 mcg	0.477
	4	4.54	3.72		
Zinc (mg)	1	11.33	4.67	Men: 11 mg	0.147
	4	10.01	4.49	Women: 8 mg	
Fiber (gms)	1	20.66	8.76	20–35 gms	0.377
	4	18.88	11.45		
Omega-3 FA (mg)	1	233.23	287.90	500 mg EPA + DHA	0.610
	4	212.54	369.05		

kcals, kilocalories; mg, milligrams; mcg, micrograms; gms, grams; FA, fatty acids; EPA, eicosapaentenoic acid; DHA, docosahexaenoic acid.

### UVB phototherapy

A small group of participants (n = 19), mostly nurses who were white, non-Hispanic females, consistently used the on-demand phototherapy device to boost serum vitamin D levels primarily during the winter months. Vitamin D levels reflected a significant increase from Baseline to 12 months (*p* < 0.001), possibly from a combination of strategies by this research team in line with recommendations from multiple sources to aim for a level of 40–60 ng/dL during the pandemic. Factors that were significant in raising serum vitamin D level at 12 months were supplemental vitamin D intake at 3 months (*p* = 0.019) and 12 months (*p* = 0.011), and duration of phototherapy treatment (*p* = 0.02).

## Discussion

This research was designed to address the vulnerabilities uncovered by previous researchers who examined the resilience of HCWs facing unrelenting challenges during daily work stressors, particularly during the pandemic. An example of published findings comes from a scoping review (Melnyk et al., 2022) on HCW wellness that included these recommendations: 1) implement individual and organizational strategies to optimize wellness for healthcare providers in areas of nutrition, exercise, mindfulness, sleep quality, and burnout mitigation, and 2) develop short-term and long-term individualized wellness and mental health interventions to address the physical and emotional tolls of COVID-19. Recognizing that both immune and cardiometabolic health deteriorate when nutrition, activity, and sleep are impaired for extended periods, the goal was to incorporate evidence-based recommendations into user-friendly, readily available resources to support busy HCWs with a desire to improve resilience through self-care and healthy lifestyle behaviors. It is important to keep in mind that the study population of highly educated, predominantly Caucasian, military-affiliated healthcare personnel limits generalizability of our results.

Activity trackers are not new or novel, yet they are a key component of research involving mHealth (mobile health), self-monitoring, and behavior change, particularly related to physical activity and sleep (Yen, 2020). Studies involving activity trackers have reported low engagement when multiple interventions are involved (Hebden et al., 2014) while others reported achieving 76%–95% wear time (Kirk et al., 2019). Similar to our study, Bentley et al. found wear time decreased over the study period, as did Gavin et al. who suggested that intervention augmentations may be needed in the study design in order to address behavioral disengagement before it affects participant success (Gavin et al., 2018; Bentley et al., 2016). Overall, our study findings are in line with the published reports of a decrease in physical activity (Melnyk et al., 2022; Palu et al., 2021; Costello et al., 2021; Tison et al., 2022) brought on by pandemic isolation, quarantine, restricted or no access to fitness centers, and longer hours at work. In fact, pre-pandemic, only 24% of U.S. adults met physical activity guidelines (Gavin et al., 2018). During in-person appointments, participants described their own challenges which are validated in their inconsistent activity data.

It was evident early on in the pandemic that poor metabolic health and unhealthy lifestyle behaviors were associated with higher risk and severity of COVID-19, even in HCWs. But few studies were published that directly evaluated key immune-enhancing nutrients vitamin C, vitamin D, zinc, fiber, and omega-3 FA in the diet during the pandemic (Story, 2021; Gombart et al., 2020; Camargo and Martineau, 2020; Grant et al., 2020; Carr and Maggini, 2017; Bonaventura et al., 2015; Cai et al., 2018). We found that our HCW population had reduced intake of all of these nutrients, except vitamin D, for the duration of the study. Early results regarding dietary intake revealed deficiencies along with excess saturated fat, sugary beverages, and calories despite individualized coaching at three timepoints coinciding with the FFQ administration, targeted health messaging on the dashboard throughout the study, and a health promotion video accessible 24/7. However, the decrease in total calories, grams of carbohydrate, and grams of saturated fat data at 12 months was reassuring and was an important teaching point for overall cardiometabolic health. DII scores did not improve significantly over time; in fact, the score at Baseline was less inflammatory than at Time 4 even with positive changes for healthy protein, carbohydrate, and fat intake. Anti-inflammatory n-3 fatty acid and fiber intake decreased over time. The HEI-2015, a secondary outcome, did not show a significant difference over time remaining at a median score of 65.2 (10.1) which is higher than a published report (Rittenhouse et al., 2021) in military soldiers 18–58 years old at 59.9 (10.2) or a matched NHANES cohort at 55.4 (3.7). Targeted health messaging failed to change usual dietary habits and low engagement with the digital dashboard may have played a role.

Sleep is often overlooked as a critical lifestyle behavior linked to immune and cardiometabolic health (Besedovsky et al., 2019; Watson et al., 2015; Centers for Disease Control and Prevention CDC, 2022a; Lloyd-Jones et al., 2022). Our sleep findings agree with most reports in the civilian community that 30% of adults achieve less than the recommended 7 or more hours of sleep each night (Centers for Disease Control and Prevention CDC, 2022a), even before the pandemic. Of note, the American Heart Association’s Construct of Cardiovascular Health was recently updated from Life’s Simple 7 to Life’s Essential 8, with the addition of sleep (Lloyd-Jones et al., 2022). Current research has demonstrated a relationship between sleep and the original 7 components of cardiovascular health; impaired sleep duration has been linked with risk factors such as BP, blood glucose, obesity, and inflammation (Lloyd-Jones et al., 2022). Another recent and underexplored relationship has been identified between vitamin D deficiency and sleep disorders defined as poor sleep quality, short sleep duration, and sleepiness (Gao et al., 2018). While our sleep data did not demonstrate significant change, the perception of many participants was that they continued to experience a decrease in sleep time, more frequent awakenings, and poor sleep quality. Correlations with blood levels of 25(OH)D warrant further study. eHealth apps, digital devices, and evidence-based strategies to facilitate sleep are widely available and should be recommended as the first line of support. Our study participants appreciated the verification of sleep disruptions via activity tracker and ISI results. Many vowed to address the issue more seriously while others sought professional medical evaluations for possible sleep apnea.

Serial body composition measurements were included with the goal of raising awareness of one’s body weight, resting energy expenditure, body mass index, lean mass, and body fat percentage. Our intent was to show the links between excess weight and fat and the higher risk for COVID-19 and cardiometabolic diseases, such as hypertension, high cholesterol, and pre-diabetes (Roskoden et al., 2017; Lichtenstein et al., 2021; Gaesser and Angadi, 2021; Mehanna et al., 2021). Obesity is linked to more than 200 chronic health conditions which contributes to employee absenteeism, retention, healthcare costs, and adverse mental health (Kyle et al., 2017; Luckhaupt et al., 2014). Luckhaupt reported that over one-third of HCWs are living with obesity; this is one of the highest rates across professions (Luckhaupt et al., 2014). Our study sample was a mix of civilian and military HCWs with body weight, body mass index, and body fat exceeding established thresholds (by gender) for about 30%–40% of the sample (see Table 5). In addition to weight challenges, the HCWs also experienced higher rates of hypertension and dyslipidemia than expected. In the early phases of the pandemic, 6% of adults with COVID-19 were HCWs and the vast majority of them had at least one underlying condition with the most common being obesity (72%), hypertension (41%), and diabetes (31%) (Kambhampati et al., 2020). Our behavioral coaching efforts were directed at these health conditions during one-on-one appointments every 3–6 months and we arranged 24/7 access to an InBody BIA machine and an automated BP machine to encourage self-monitoring. Raising awareness of even minor negative changes in body composition, blood lipid levels, blood pressure, poor sleep habits, unhealthy eating, and inadequate amounts of physical activity, was called “eye-opening” and “motivating” by participants. Incorporating exploratory gene expression analyses into this study offered additional appeal for participants and introduced a paradigm shift in thinking about one’s ability to overcome genetic predisposition through healthful nutrition, regular physical activity, and restful sleep.

The opportunity to extend the study by 6 months allowed for additional in-person assessments and recommendations based on changes in the participant immune and cardiometabolic profile. Only recently have an abundance of mostly short-term studies related to HCWs and the impact of the pandemic on their physical and mental health been published (Hassamal et al., 2021; Institute for Healthcare Improvement IHI, 2020). Findings from this study have been consistent with research results describing the pandemic’s effect on lifestyle behaviors (Hassamal et al., 2021; Shreffler et al., 2020), specifically nutrition (Deschasaux-Tanguy et al., 2021; Merino et al., 2021) and activity (Melnyk et al., 2022; Deschasaux-Tanguy et al., 2021).

### Limitations

Due to the decision to recruit from only one medical center and to enroll both military and civilian HCWs, the sample is not representative of community participants during the pandemic. Our selection of thresholds for activity, dietary intake, and sleep were evidence-based yet may not be universally accepted and appear to change over the years as new research findings are published and adult lifestyle habits change. The activity tracker used for this study was an early model that is no longer available and provided only basic information including daily steps, distance, intensity of activity, minutes of sleep, and sleep awakenings. Participants were reminded to charge and sync the Fitbit with their phone app on a regular basis but this led to gaps in data recording. The majority of participants admitted that they chose not to access the interactive dashboard on more than one or two occasions as it required too many steps or was time consuming. It was not possible to electronically track logins or time spent viewing the dashboard, and self-report data are known to be unreliable. This was a missed opportunity because personalized, educational messaging was created and distributed using the dashboard. This led to uncertainty surrounding the “dose” of the health coaching received by each participant.

An observational study is inherently less rigorous and subject to concerns of internal and external validity due to the lack of control over the level of engagement with toolkit components or exposure to health information. There are several confounding elements including a more educated sample population, shift work, multiple stressors related to the ongoing pandemic, geographic location influencing vitamin D levels, underlying health conditions, and a potential lack of familiarity with activity tracker devices and web-based portals.

## Conclusion

The study population exhibited similar alterations in lifestyle behaviors as the general community in part due to the pandemic. Early enthusiasm by HCWs to accept responsibility for their declining metabolic health and increasing risk for cardiovascular disease was short-lived. The HCWs expressed appreciation for alerting them to long-neglected health promoting behaviors and self-care strategies. More research focused on HCW physical and mental health is urgently needed. These investigations must include innovative and emerging technologies that can examine crosstalk between gut health, immune status, and lifestyle behaviors yielding personalized recommendations for HCWs. Care for the caregiver has never been more important or necessary to restore a sense of wellbeing, reduce risk for cardiometabolic disease, and promote resilience.

## Data Availability

The raw data supporting the conclusions of this article will be made available by the authors, without undue reservation.
